# Prevalence and severity of fatigue in treated hypothyroidism: results of a UK survey

**DOI:** 10.1530/ETJ-25-0044

**Published:** 2025-05-14

**Authors:** Lydia Grixti, Holly Fisher, Julia Priestley, Cheryl McMullan, Anna Woollven, Petros Perros, Anna Louise Mitchell, Earn H Gan, Simon H Pearce

**Affiliations:** ^1^ Translational and Clinical Research Institute, Newcastle University, Newcastle-upon-Tyne, UK; ^2^ Endocrine Unit, Royal Victoria Infirmary, Newcastle-upon-Tyne, UK; ^3^ Population Health Sciences Institute, Faculty of Medical Sciences, Newcastle University Faculty of Medical Sciences, Newcastle upon Tyne, UK; ^4^ British Thyroid Foundation, Harrogate, UK

**Keywords:** Hashimoto thyroiditis, hypothyroidism, fatigue, tri-iodothyronine, levothyroxine, quality of life, FACIT-F

## Abstract

**Background:**

A substantial proportion of patients taking thyroid hormone replacement for hypothyroidism show persistent symptoms. We sought to explore the prevalence and degree of fatigue in this patient group.

**Methods:**

An online survey including the FACIT-F fatigue scale was distributed by two UK patient support organisations, the British Thyroid Foundation (BTF) and The Thyroid Trust (TTT). Overall, 1,334 responses were received, of which 1,251 were complete, unique and from patients with primary hypothyroidism/Hashimoto thyroiditis who reported taking thyroid hormone replacements.

**Results:**

Ninety eight percent of respondents were women and the mean duration of treatment was 10.8 years (SD: 9.74). The mean fatigue score on the FACIT-F scale was 20.5 (SD: 10.5), with 89% of respondents fulfilling criteria for abnormal fatigue. Fatigue scores were not significantly different between respondents of different ages, gender, treatment type or treatment duration. FACIT-F scores were positively correlated with self-declared overall health state (Pearson *r* = 0.576, *P* < 0.001).

**Conclusions:**

Fatigue in treated hypothyroidism is very common, and the FACIT-F scores reported are comparable or worse than those recorded for many other chronic conditions. This study suggests that addressing fatigue in this patient group will be key to improving wellbeing and quality of life.

## Introduction

Hashimoto thyroiditis is the commonest autoimmune disease, with an estimated incidence of 160 to 210/100,000 per year in the UK (1). While it may remain asymptomatic for many years during which it is manifest only as detectable serum thyroid peroxidase antibodies (TPOAb) or a heterogeneous hypoechoic pattern on thyroid ultrasound, in around 50% of people it progresses to hypothyroidism over around 20 years (2). Management of hypothyroidism should be straightforward in principle, with replacement of endogenous thyroid hormones with oral levothyroxine (LT4) medication (3). However, over a number of years it has become apparent that not all patients taking levothyroxine feel that it fully restores their wellbeing (4, 5, 6). Persistent symptoms, psychological morbidity and dissatisfaction with levothyroxine treatment have been documented in more than 10% of patients from several different healthcare settings including the UK, USA and Netherlands (4, 5, 6). Perplexingly, persistent symptoms and dissatisfaction with levothyroxine treatment do not correlate with reduced serum tri-iodothyronine (T3) concentrations in patients treated with LT4 monotherapy (7, 8), nor have multiple randomised controlled trials of adding T3 to levothyroxine as combination therapy shown benefit in terms of symptoms, cognition or quality of life (8, 9, 10, 11). On a larger population level, two recent online patient surveys have also shown ‘prominent dissatisfaction’ with thyroid hormone treatment among hypothyroid patients, although there was limited analysis of the nature of symptoms and their interactions with factors other than current treatment (12, 13). During a small informal survey in which hypothyroid patients were asked to rank the severity of their symptoms, we observed that fatigue was rated as the most troublesome symptom for most patients (>70%), and the dominance of fatigue and tiredness compared to other persistent symptoms has been confirmed by other investigators (14, 15, 16). Nevertheless, fatigue has also been demonstrated to improve in many hypothyroid patients following initiation of levothyroxine replacement (15, 17).

Fatigue has been defined as ‘a sense of physical tiredness and lack of energy, distinct from sadness or weakness’ (18). It can be a normal experience in healthy people and distinguishing normal or expected fatigue following different levels of activity from abnormal or ‘pathological’ fatigue is not straightforward. Previous studies in Dutch and Mexican hypothyroid patients have quantified fatigue using the multidimensional fatigue inventory (MFI-20) and the fatigue severity scale (14, 17), respectively. In order to define the prevalence of abnormal fatigue more clearly, we embarked on this large survey of UK hypothyroid patients using the validated FACIT-F (Functional Assessment of Chronic Illness Therapy-Fatigue) questionnaire (19), which quantifies the functional impact of chronic illness, and cognitive, emotional and physical aspects of fatigue.

## Subjects and methods

### Survey design and rollout

The survey was released online via the website and social media networks of two UK-based patient support organisations, the BTF (www.btf-thyroid.org) and TTT (www.thyroidtrust.org), between May 29th and June 30th 2024. Respondents were first asked to confirm that they were over 18 years of age and had a diagnosis of primary hypothyroidism/Hashimoto thyroiditis. The next section of the survey was the Functional Assessment of Chronic Illness Therapy-Fatigue (FACIT-F) questionnaire (Supplementary Table 1 (see section on Supplementary materials given at the end of the article)), which is a 13-item instrument that has been widely used to quantify the effects of fatigue in a variety of chronic diseases including cancer, anaemia, rheumatoid arthritis and inflammatory bowel disease (19, 20, 21). The 13 questions cover different aspects of fatigue including tiredness, weakness, somnolence and the effects of fatigue on daily activities including eating, socialising and the need for assistance. It is scored on a Likert scale (0–4), with a maximum score of 52 indicating no fatigue. The mean score in a healthy population is 43 (±17 SD) (22), with a score of 34 or less considered diagnostic for abnormal fatigue (23). The next item asked the respondents to rate their own health today, on an ordinal scale of 0–10 with prompts for zero being ‘worst possible health state’ and ten being ‘best possible health state’. Respondents were next asked to use a free text box to answer the question ‘other than tiredness or fatigue, what do you consider the most troublesome symptom of your thyroid underactivity’? After review of the breadth of responses, these additional symptom responses were categorised into the following groups: cognitive problems (including brain fog), constipation, gastrointestinal symptoms, hair or nail problems, headache, insomnia, mood disturbance or anxiety, musculoskeletal pain or stiffness, none, palpitations, skin problems, temperature regulation issues (including feeling cold) and weight management issues. The remaining questions asked about what medications were being taken for hypothyroidism, age (18–50 or >50 years), gender and duration of treatment for hypothyroidism. The survey engine was set so that participants had to respond to each question before seeing the next one. Responses were anonymous and participants consented for the use of these data. The Newcastle University Research Ethics Committee gave approval for the survey (ref: 47712/2023).

### Responses

Overall, the survey received 1,334 responses over the 33 days in which it was open. One hundred and twenty-six responses were from the link shared by the TTT and 1,208 from the link shared by the BTF to its members and followers. The BTF link had a total of 2,569 clicks, indicating a 47% completion rate. However, as the first question asked respondents to confirm that they were over 18 and had a diagnosis of primary hypothyroidism/Hashimoto thyroiditis, an unknown number of these clicks are likely to have been made by people outside these specifications who then did not go on to complete the survey.

**Figure 1:**
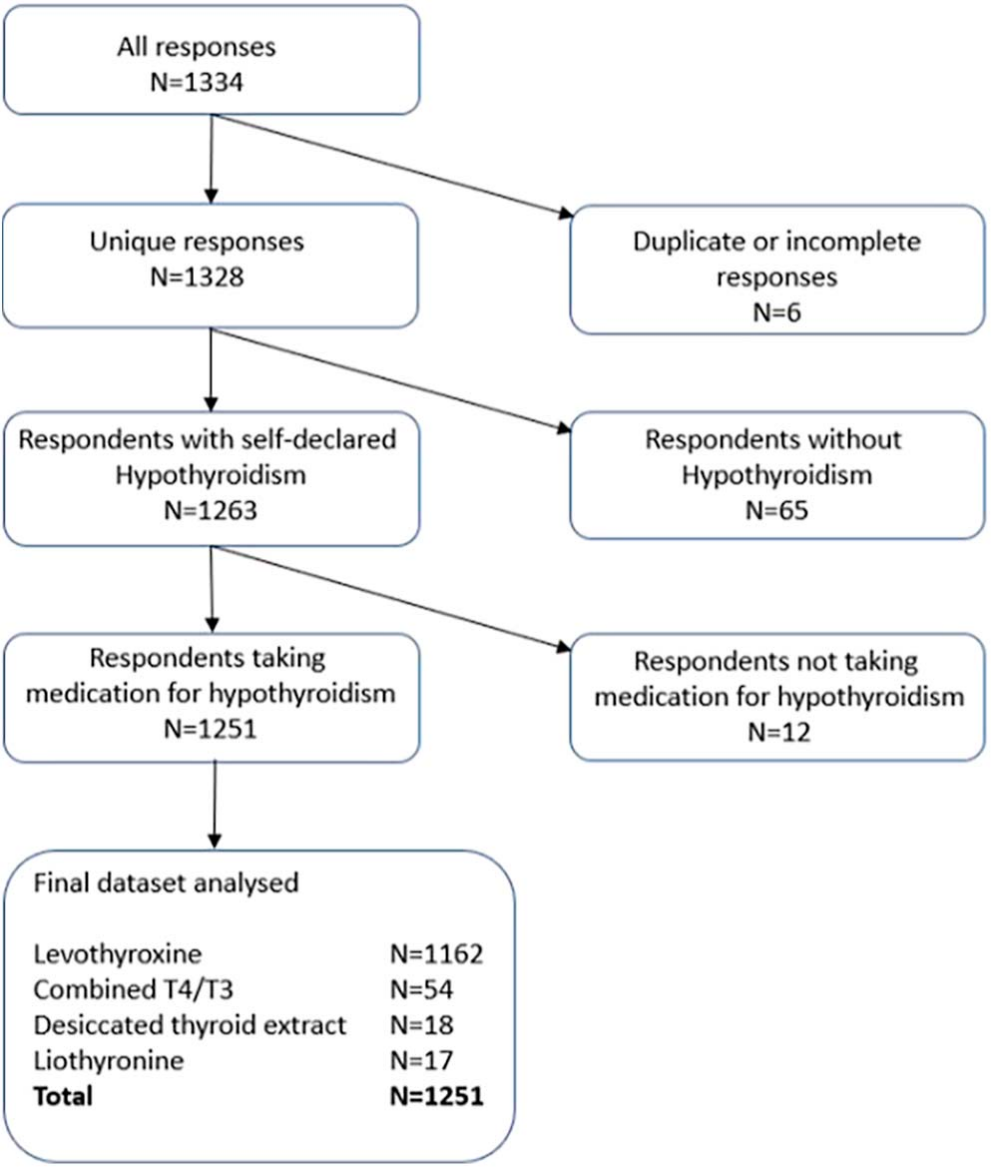
Survey response flowchart. *n* = number of responses. Total responses were 1,334. Final dataset analysed were 1,251 responses, following elimination of duplicates, incomplete responses, respondents without a diagnosis of primary hypothyroidism and respondents not taking thyroid hormone replacement. Thyroid hormone replacement included levothyroxine (T4) (*n* = 1,162), combined levothyroxine and liothyronine (T4/T3) (*n* = 54), DTE (*n* = 18) and liothyronine alone (*n* = 17).

Out of the total number of 1,334 responses, five were excluded for being duplicates and one person was excluded due to not answering all demographic questions. Sixty-five participants declared either that they did not have a diagnosis of primary hypothyroidism (*n* = 17) or that they were unsure (*n* = 48), and these were also excluded. An additional 12 responses were excluded as they stated that they were taking no medication for hypothyroidism. This left 1,251 responses which were taken as a population with self-declared, treated primary hypothyroidism/Hashimoto thyroiditis for further analysis (Fig. 1).

### Analysis

Multivariate linear regression was used to assess the effect of demographic covariates on FACIT-F scores. Age, gender, treatment and duration of treatment for hypothyroidism, and patient support organisation were included in the model as independent variables. Graphical methods were used to check that the regression assumptions were satisfied. The correlation between FACIT-F scores and overall health state was assessed using Pearson’s correlation coefficient. Analyses were carried out using GraphPad-PRISM version 10.2.3 and R version 4.2.2.

## Results

**Figure 2:**
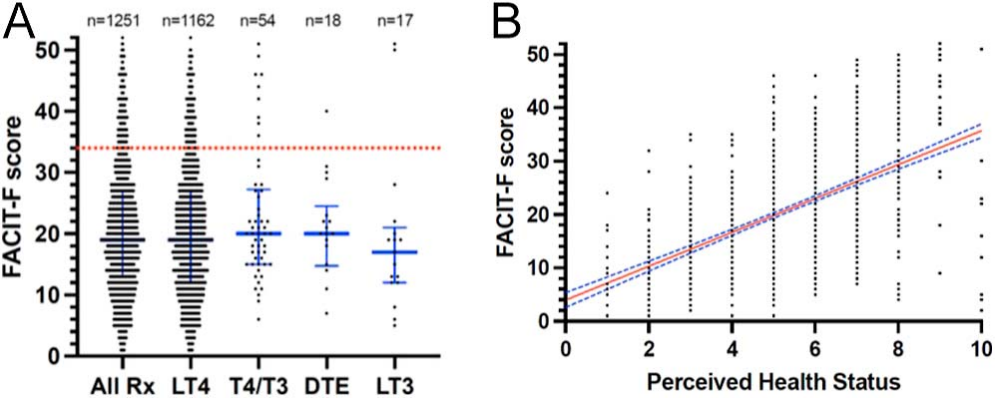
(Panel A): the distribution of FACIT-F scores according to thyroid hormone replacement received. All respondents taking any formulation of thyroid hormone replacement (*n* = 1,251), those on levothyroxine (LT4) *n* = 1,162, combined levothyroxine (T4) and liothyronine (T3) *n* = 54, DTE *n* = 18 and liothyronine (LT3) *n* = 17. No significant difference is present. Blue lines and error bars are median ± IQR; red dotted line is the threshold (34) for declaring abnormal fatigue (23). (Panel B): the respondents’ perception of overall health state (*n* = 1,251), scored using an ordinal scale of 0–10 with zero being ‘worst possible health state’ and ten being ‘best possible health state’, showing moderate positive correlation with FACIT-F scores (Pearson *r* = 0.576, *P* = < 0.001). Red line shows linear regression, with 5–95% confidence intervals in blue dashed lines.

Of the 1,251 responses included in the analysis, 1,227 (98%) were from women and the mean duration of thyroid hormone replacement therapy was 10.8 years (SD: 9.74) (range: 1 month to 57 years). The mean fatigue score on the FACIT-F scale was 20.5 (SD: 10.5) with 89% (1,112 of 1,250) reporting a score of 34 or less, indicating abnormal fatigue (23) (Table 1). Duration of therapy was not correlated with FACIT-F score (Pearson *r* = 0.0031, *P* = 0.913) (Supplementary Fig. 1). The distribution of FACIT-F scores according to thyroid hormone replacement received is shown in Fig. 2A. There was no statistical difference between treatment groups (Table 1).

**Table 1:** FACIT-F survey data in treated primary hypothyroidism. Survey results showing differences in FACIT-F scores according to gender, age, different treatments and source of respondent.

	*n* (%)	FACIT-F score, mean (SD)	FACIT-F score, median (IQR)	Abnormal fatigue (%)^†^	Regression coefficient (95% CI)	*P*-value*
All responses	1,251	20.5 (10.5)	19 (13–27)	1,112 (88.96)	-	
Gender						
Female	1,227 (98.1)	20.4 (10.5)	19 (13–27)	1,093 (89.1)	(Reference)	
Male	23 (1.8)	23.6 (11.6)	24 (15–28)	19 (82.6)	3.3 (−1.0, 7.6)	0.134
Other	1 (0.1%)	NA	NA	1 (100%)	NA	
Age, years						
18–50	673 (53.8)	19.9 (10.0)	19 (12–26)	611 (90.8)	(Reference)	
>50	578 (46.2)	21.1 (11.1)	20 (13–27.8)	502 (86.9)	1.2 (−0.0, 2.5)	0.050
Treatment						
Levothyroxine monotherapy	1,162 (92.9)	20.4 (10.5)	19 (12–27)	1,035 (89.15)	(Reference)	
Combined T4/T3	54 (4.3)	22.9 (10.9)	20 (15.3–27)	45 (83.3)	2.3 (−0.56, 5.2)	0.114
DTE	18 (1.4)	20.1 (9.19)	20 (15.8–22.8)	17 (94.4)	−0.76 (−5.7, 4.1)	0.760
Liothyronine monotherapy	17 (1.4)	19.4 (13.1)	17 (12–20)	15 (88.2)	−2.4 (−7.5,2.7)	0.362
Patient support organisation						
British Thyroid Foundation	1,137 (90.9)	20.2 (10.4)	19 (12–26)	1,017 (89.4)	(Reference)	
The Thyroid Trust	114 (9.1)	23.6 (11.2)	22.5 (15.3–30.8)	96 (84.2)	3.3 (1.3, 5.4)	0.001
Treatment duration in years	10.8 (9.74)^‡^	NA	NA	NA	−0.03 (−0.09, 0.04)	0.407

* Results of multivariate linear regression with FACIT-F score as the dependent variable. For each categorical variable, the reference category is indicated in the table.

^†^ Abnormal fatigue was defined as FACIT-F score ≤34 (ref. 23).

^‡^ Value is mean (SD).

DTE, desiccated thyroid extract.

FACIT-F scores according to various covariates are presented in Table 1 along with the results of fitting a multivariate linear regression model. There was no evidence that FACIT-F scores are influenced by gender, age, treatment type or treatment duration. However, FACIT-F scores in patients from the Thyroid Trust were on average 3.3 points higher than those from the British Thyroid Foundation (95% CI (1.3, 5.4), *P* = 0.001). Demographic variables split by patient organisation are shown in Supplementary Table 2.

In addition to FACIT-F, respondents were asked to rate their overall health state with the question ‘please indicate how good or bad your health state is today, in your opinion’. This perception of health was moderately positively correlated with FACIT-F (Pearson *r* = 0.576, *P* < 0.001) (Fig. 2B).

**Figure 3:**
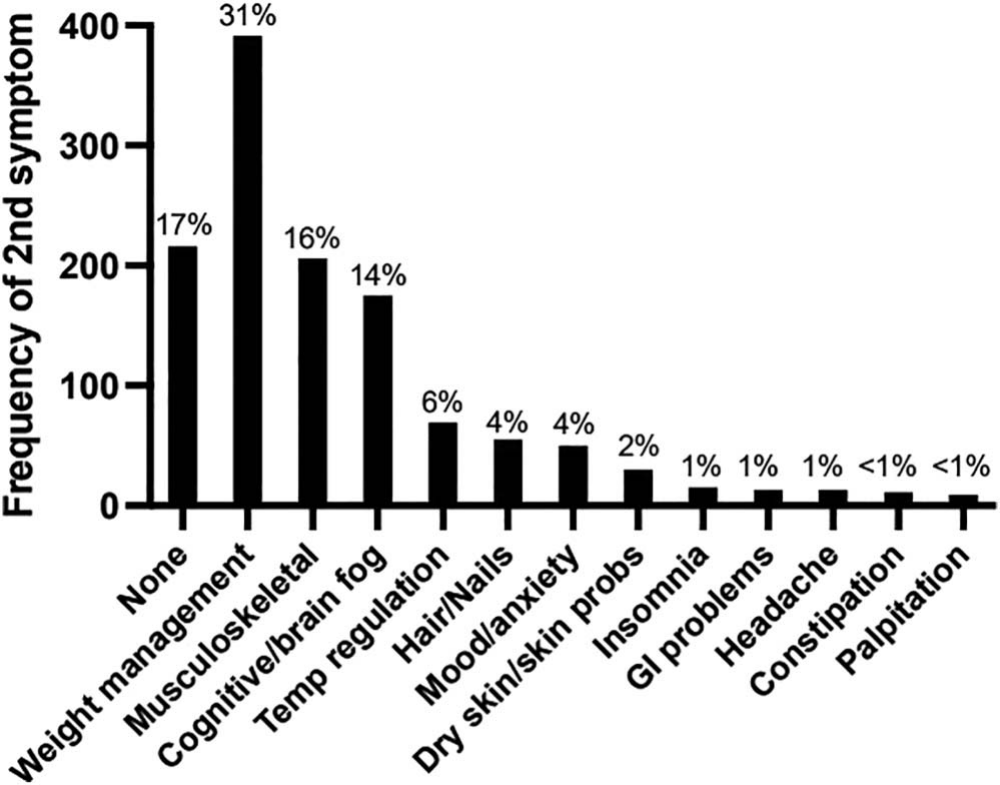
Survey results of second most troublesome additional symptom, after fatigue. Data represented as percentage of total (*n* = 1,251).

Finally, participants were asked to name their most troublesome symptom other than fatigue. Seventeen percent responded that they had no other symptom. The other frequent responses were weight management problems (31%), musculoskeletal aches and pains (16%) and cognitive problems or brain fog (14%). The remaining 22% of respondents had a variety of complaints which are shown in Fig. 3.

## Discussion

Although persistent symptoms and dissatisfaction among patients treated for hypothyroidism/Hashimoto thyroiditis have been recognised for many years, there has been limited exploration of the nature of these persisting symptoms (4, 5, 6, 14, 15, 16, 17). Our study shows that the majority of respondents had significant fatigue as judged by the validated FACIT-F instrument, the mean score of 20.5 (SD 10.5) indicating similar or worse fatigue than patients with a variety of other common chronic conditions (Table 2). A study of 254 patients with active systemic lupus erythematosus (SLE) showed a mean FACIT-F score of 19.1 (SD: 11.5) which improved to 24.8 (SD: 13) following 12 weeks of immunosuppressive treatment (24). Compared to other chronic conditions, our respondents with primary hypothyroidism/Hashimoto thyroiditis reported more severe fatigue than anaemic patients with cancer (mean score 24), ambulatory patients with inflammatory bowel disease (median score 38), Parkinson’s disease (mean score 34) and rheumatoid arthritis (mean score 29) (see Table 2 for other comparators) (20, 22, 24, 25, 26, 27, 28, 29).

**Table 2:** A comparison of FACIT-F scores in different chronic conditions. Validated FACIT-F scores as used in other studies for other chronic conditions, including SLE, anaemic and non-anaemic cancer patients, end-stage renal disease (ESRD) on dialysis, chronic obstructive pulmonary disease (COPD), ambulatory inflammatory bowel disease patients (IBD), Parkinson’s disease and rheumatoid arthritis. Hashimoto thyroiditis shows the second worst FACIT-F score on the list, second to untreated SLE (20, 22, 24, 25, 26, 27, 28, 29).

Condition	Reference	Sample size, *n*	FACIT-F score, mean ± SD
Hypothyroidism/Hashimoto thyroiditis (on treatment)	This study	1,252	20.5 ± 10.5
SLE	24	254	19.1 ± 11.5
Anaemic cancer patients	22	2,292	24 ± 12.6
Non-anaemic cancer patients	22	113	40 ± 9.8
Ambulatory cancer patients	27	335	34.63 ± 10.10
ESRD, on dialysis	29	46	41.1 ± 9.9
COPD	28	434	46 (38–50)*
Ambulatory IBD	25	202	38 (1–52)*
Parkinson disease	26	118	34.2 ± 9.9
Rheumatoid arthritis	20	631	29.17 ± 11.06

* Value is median (IQR).

Although we have not performed a formal validation study of FACIT-F in patients with Hashimoto thyroiditis, the close correlation between the fatigue scores and self-rated health state (Fig. 2B) suggests that fatigue is a major contributor to persistent symptoms in this patient group. Indeed, when asked about additional symptoms, 17% of respondents did not have other symptoms. Previous studies have shown that fatigue, tiredness and lack of vitality are dominant symptoms in people with hypothyroidism (14, 15, 16, 17). Winther and coworkers studied 68 patients with newly diagnosed hypothyroidism, and tiredness was the symptom that was most over-represented compared to the healthy population (17). Furthermore, following 6-months’ treatment with levothyroxine, tiredness and vitality remained impaired according to the ThyPRO36 and SF-36 scales (17). Similarly, Hidalgo *et al.* have recently demonstrated that fatigue was the most frequent symptom during follow-up of a freshly treated cohort of hypothyroid patients, reported in around 40% (16). These studies along with our current survey point to fatigue as being the major target to focus on in order to improve the wellbeing of this patient group.

In common with previous surveys of patients with hypothyroidism (12, 13), we did not find clinically relevant differences in fatigue score or self-reported health status between patients taking levothyroxine monotherapy and those using other forms of thyroid hormone replacement (Fig. 2A). This is not surprising, given that numerous RCTs have demonstrated no benefit in terms of wellbeing or quality of life in people treated with T4/T3 combination therapy compared to those treated with levothyroxine monotherapy (8, 9, 10, 11). In addition, our study had low numbers of respondents using tri-iodothyronine monotherapy (*n* = 17) or desiccated thyroid extract (DTE) (*n* = 18), therefore we cannot draw firm inferences about these groups.

That 89% of our respondents fulfil criteria for abnormal fatigue emphasises that this is a patient group with chronic ill health, and this appears to be largely independent of their thyroid hormone replacement (Fig. 2A). Several alternative hypotheses have been proposed to explain persistent fatigue in this patient group, including physical factors such as persistent chronic autoimmunity to the thyroid despite adequate hormone replacement, co-existing non-thyroid autoimmune disorders, or other non-autoimmune physical health comorbidities (30, 31, 32). In addition, psychological factors have also been suggested, including that persistent symptoms reflect the burden of living with a chronic illness, or that symptoms reflect an underlying somatising disorder or pessimistic personality type (type D personality) (33, 34). In support of the persistent autoimmunity hypothesis, a systematic review showed that thyroid peroxidase antibodies were found to correlate with persisting symptoms in 16 of 23 studies analysed (31).

Along with fatigue, respondents mentioned weight management problems, musculoskeletal aches and pains, and cognitive problems/brain fog as being their major concerns (Fig. 3). The relationship between thyroid conditions and body weight is complex. The recent Health Survey for England (2021) documented that 26% of the English population are obese, with a further 38% being overweight (in total, 64% either overweight or obese). Therefore, the proportion of respondents who complained of weight management problems (31%) is likely representative of the general population, irrespective of thyroid status (35). It is also acknowledged that fluctuations in weight even within the accepted normal BMI can still be a cause for distress (36). Musculoskeletal and cognitive symptoms were also common and could represent manifestations of hypothyroidism that may be amenable to other specific treatments such as targeting autoimmunity. On the other hand, they may similarly be representative of the background population, with one third of the UK population known to be living with a musculoskeletal condition (37). In addition, it is estimated that one third of the UK female population are experiencing symptoms related to perimenopause or menopause, which are commonly implicated for changes in cognition and brain fog (38). However, our data do not support this hypothesis, as older people (≥50 years) did not show worse fatigue scores than younger respondents. Furthermore, detailed research is required to understand why some of the typical clinical features of hypothyroidism such as constipation and dry skin (Fig. 3) seem to be relieved more commonly than these other manifestations following treatment. Furthermore, the contribution of non-thyroid comorbidity leading to persistent symptoms needs to be explored too, as mentioned above.

The strength of this survey is the large number of responses and the positive correlation of respondents’ health status with the FACIT-F fatigue score. In addition, there were no important differences between the responses of participants who were members of the two different support organisations (Suppl Table 2). Weaknesses include that the diagnosis of primary hypothyroidism/Hashimoto thyroiditis is self-reported and cannot be validated. In addition, we did not collect information about the biochemical control of the respondents’ hypothyroidism, therefore it is possible that suboptimal thyroid hormone replacement may be a contributing factor to persisting symptoms. As we were interested in menopausal status but conscious not to gather potentially identifying information, data about age were collected in a binary way, with people being grouped as 18–50 or over 50 years. This resulted in our analysis lacking power compared to ascertaining age as a continuous variable. Primary hypothyroidism/Hashimoto thyroiditis shows a marked female preponderance; however, even accounting for that, male responders were under-represented in our survey, with only 23 men (1.8%) responding. A lack of responses from men was also noted on the previous two online surveys, including another from the UK and one conducted in the USA (12, 13). Carlé and coworkers have shown that men present with very similar symptoms of hypothyroidism to women, but that serum TSH is higher at diagnosis, suggesting a higher threshold for thyroid function testing in men (39). Conversely, it has been demonstrated that women have more symptoms than men in several other conditions, as well as in the general population (40). For our survey, it may also be argued that men with hypothyroidism simply access forms of support other than patient support organisations, and so were not aware of the survey. Finally, respondents to an online survey promoted by patient support organisations may not be representative of the whole population with hypothyroidism taking thyroid hormone replacement therapy. Participants may be more motivated to respond because they feel unwell and thus these responses may bias results and overestimate the overall burden of illness. A future survey could helpfully correlate FACIT-F scores directly with measured serum TSH, and consider targeting men with hypothyroidism perhaps by recruiting directly from primary care or endocrinology services rather than through patient support organisations.

Overall, this survey shows that fatigue in people with treated hypothyroidism is very common and associated with substantial functional impairments when acknowledging the FACIT-F scores as being comparable or worse than those recorded for many other chronic conditions. Fatigue was not associated with duration of treatment or type of thyroid hormone replacement taken, shifting the focus of care away from thyroid hormone replacement towards other treatment modalities. Addressing fatigue as the dominant symptom in this patient group will be key to improving wellbeing and quality of life.

## Supplementary materials



## Declaration of interest

PP discloses honoraria from IBSA (Institut Biochimique SA). SHP receives speaker fees from IBSA, Merck, Immunovant and is a consultant for Immunovant and Lycia.

## Funding

This study was funded by the Medical Research Councilhttps://doi.org/10.13039/501100000265, UK, grant ID MR/Z503617/1. Data are available on request from the corresponding author.
