# Supplementary material for: Prevalence and severity of fatigue in treated hypothyroidism: results of a UK survey
Source: Eur Thyroid J. 2025 May 14;14(3):e250044. doi: 10.1530/ETJ-25-0044 (PMC12084796; doi:10.1530/ETJ-25-0044)
Supplement: Supplementary file 1 [file supplementary_materials.pdf]

Supplementary Figure 1: FACIT-F score correlation with duration of treatment

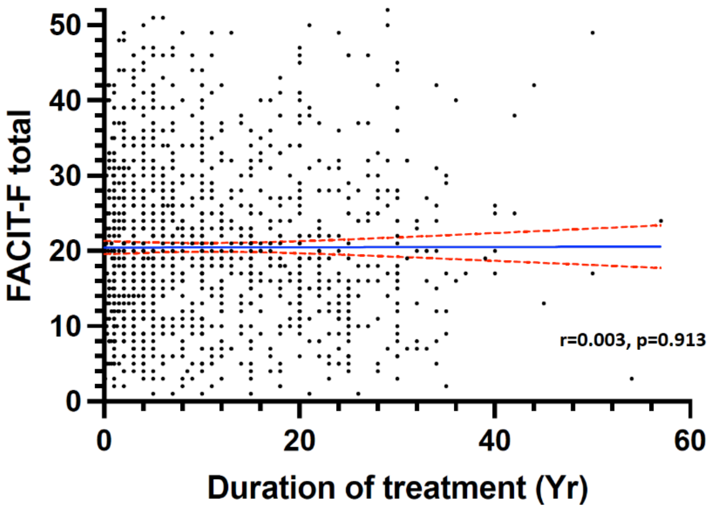

Supplementary Figure 1| Pearson correlation of duration of treatment in years with FACIT-F score from filtered responses (N=1251). No correlation demonstrated (Pearson r=0.003, p=0.913).

Supplementary Figure 2: Distribution of FACIT-F scores

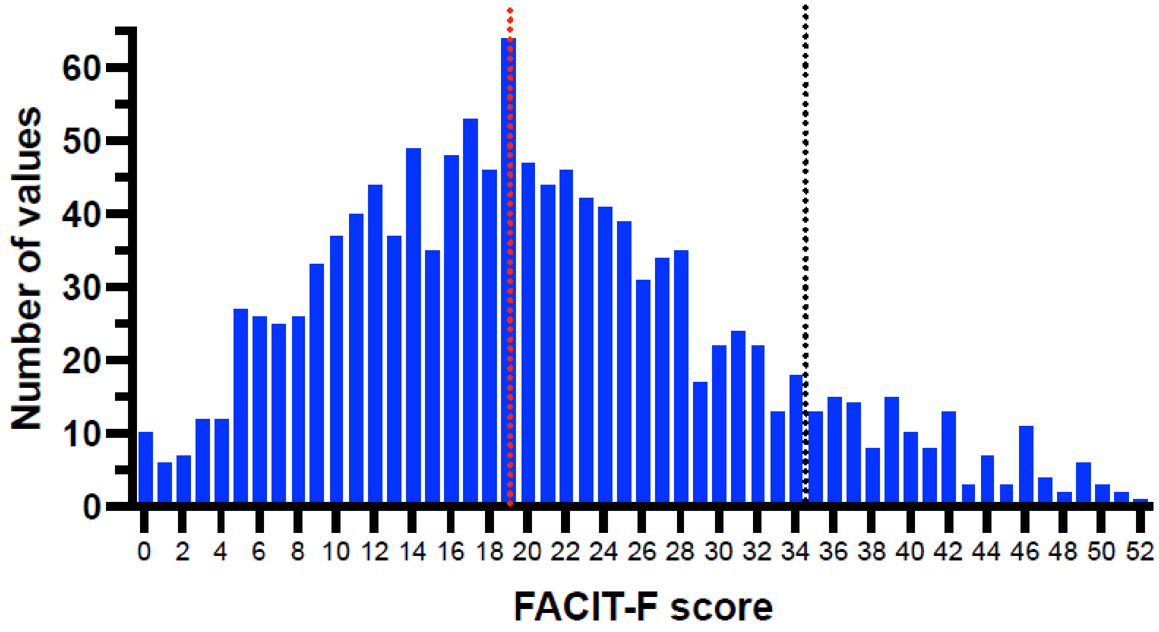

Supplementary Figure 2 | Distribution of median FACIT-F scores for analysed patient responses (N=1251). Plotted as raw source data. Red dotted line showing median score of 19. Black dotted line showing threshold for abnormal fatigue ≤34 (ref 23).

Supplementary Table 1: Study Survey Questionnaire

**Supplementary Table 1: Survey Questionnaire with general questions and FACIT-F Scale Questionnaire**
1) I have a diagnosis of primary or autoimmune hypothyroidism/Hashimoto thyroiditis<br>**Yes/No**
2) Please let us know more about you: Your age<br>**I am aged 18-50 years, I am more than 50 years of age, Rather not say**
3) Please let us know more about you: Gender- How do you identify?<br>**Woman, Man, Non-binary, Rather not say**
4) (FACIT- F Scale Questionnaire) (Likert Scale) **4-0**<br>I feel fatigued<br>I feel weak all over<br>I feel listless ("washed out")<br>I feel tired<br>I have trouble starting things because I am tired<br>have trouble finishing things because I am tired<br>I have energy (-)<br>I am able to do my usual activities (-)<br>I need to sleep during the day<br>I am too tired to eat<br>I need help doing my usual activities<br>I am frustrated by being too tired to do the things I want to do<br>I have to limit my social activity because I am tired
5) Other than tiredness or fatigue, what do you consider the most troublesome symptom of your thyroid underactivity? *(free text)*
6) Please indicate on this scale how good or bad your own health is today in your opinion: **0-10**<br>*(0 being the worst possible health state and 10 being the best possible health state)*
7) Please let us know what medication you are taking for hypothyroidism: *Choice from:*<br>**Levothyroxine (T4), Tri-iodothyronine (T3, Liothyronine), Combined Levothyroxine and Liothyronine (T4/T3), Desiccated thyroid extract (DTE, 'Natural' or 'Armour') or No medication**
8) How long is it since you started treatment for hypothyroidism? **(Please reply in years)**

Supplementary Table 1| Survey Questionnaire including FACIT-F Scale Questionnaire (Version 4). FACIT-F refers to the Functional Assessment of Chronic Illness Therapy 13-item Fatigue subscale. Responses given concern the previous 7 days. Scored on a Likert scale 4-0 where 4 is 'Not at all', 3 is 'A little bit', 2 is 'Somewhat', 1 is 'Quite a bit' and 0 is 'Very much' except for 2 questions which are scored negatively, marked as (-). The calculated final score ranges from 0-52. The higher the score, the better the quality of life. Abnormal fatigue was defined as FACIT-F score ≤34 (ref 23).

Supplementary Table 2: A comparison of demographic details and treatment between the British Thyroid Foundation and The Thyroid Trust.

	**British Thyroid Foundation** (N = 1137)	**The Thyroid Trust** (N=114)
**Gender, n (%)**		
Female	1115 (98.1%)	112 (98.2%)
Male	21 (1.8%)	2 (1.8%)
Other	1 (0.1%)	0 (0%)
**Age, n (%)**		
18-50yr	627 (55.1%)	46 (40.4%)
>50yr	510 (44.9%)	68 (59.6%)
**Treatment, n (%)**		
Levothyroxine monotherapy	1067 (93.8%)	95 (83.3%)
Combined T4/T3	44 (3.9%)	10 (8.8%)
Desiccated thyroid extract	16 (1.4%)	2 (1.8%)
Liothyronine monotherapy	10 (0.9%)	7 (6.1%)
**Treatment duration in years**		
Mean (SD)	10.4 (9.54)	14.4 (11.0)
Median (IQR)	7.00 (3.00,16.0)	12.0 (5.25,22.0)
**FACIT-F Score**		
Mean (SD)	20.2 (10.4)	23.6 (11.2)
Median (IQR)	19.0 (12.0,26.0)	22.5 (15.3,30.8)
Abnormal, n(%)*	1017 (89.4%)	96 (84.2%)

Supplementary Table 2| Survey results showing differences in patient demographics and treatment between patient organisations British Thyroid Foundation (BTF) and The Thyroid Trust (TTT). *Abnormal fatigue was defined as FACIT-F score ≤34 (ref 23).
